# Comparative analysis of coronal balance with clinical correlation between degenerative and isthmic lumbar spondylolisthesis: a retrospective study

**DOI:** 10.3389/fmed.2026.1902310

**Published:** 2026-10-23

**Authors:** Hengren Li, Chen Liu, Xiaodong Liu, Heng Yang, Xiaoyang Liu, Fei Jia, Xingang Cui

**Affiliations:** 1 Department of Spine Surgery, Shandong Provincial Hospital, Shandong University, Jinan, China; 2 Department of Spine Surgery, Shandong Provincial Hospital Affiliated to Shandong First Medical University, Jinan, China

**Keywords:** degenerative spondylolisthesis, isthmic spondylolisthesis, coronal balance, segmental wedge angle, decision tree

## Abstract

**Objective:**

Few studies have examined the relationship between lumbar spondylolisthesis and coronal balance. This study compared coronal radiographic parameters between degenerative spondylolisthesis (DS) and isthmic spondylolisthesis (IS) and investigated their associations with clinical characteristics.

**Methods:**

We retrospectively reviewed 150 patients with single-level lumbar spondylolisthesis and persistent symptoms after at least 3 months of conservative treatment. Standing whole-spine anteroposterior radiographs were used to measure clavicle angle, T1 tilt, pelvic coronal tilt, segmental wedge angle, spondylolisthesis vertebral tilt angle, lateral vertebral translation, lumbar Cobb angle, lumbar coronal balance, and C7 plumb line deviation from the central sacral vertical line. Demographic, clinical, and radiographic variables were compared between groups. Multivariable logistic regression identified factors distinguishing DS from IS. A decision tree assessed whether segmental wedge angle predicted lower limb pain distribution in DS.

**Results:**

Patients with DS were older, whereas those with IS were taller and heavier. Segmental wedge angle, spondylolisthesis vertebral tilt angle, and lumbar coronal balance were greater in the DS group than in the IS group (2.7 ± 1.9° vs. 1.8 ± 1.7°, *P* = 0.005; 4.7 ± 3.6° vs. 3.5 ± 2.4°, *P* = 0.016; and 10 ± 7 mm vs. 5 ± 3 mm, *P* < 0.001, respectively). Segmental wedge angle and lumbar coronal balance independently distinguished DS from IS. The DS group had higher leg-pain VAS scores (6.0 ± 1.1 vs. 4.6 ± 1.6, *P* < 0.001), but back-pain VAS and ODI scores did not differ between groups. In patients with DS and a segmental wedge angle of 1.3° to <5.0°, lower limb pain was associated with wedge orientation and occurred mainly on the concave side. Pain distribution was not predictable from wedge orientation when the angle was <1.3° or ≥5.0°.

**Conclusion:**

DS is associated with greater lumbar coronal imbalance and segmental wedging than IS. Segmental wedge angle and lumbar coronal balance may help distinguish these conditions. In DS, wedge orientation may also indicate the side of lower limb pain when the segmental wedge angle is between 1.3° and <5.0°.

## 1 Introduction

According to the Wiltse-Newman classification system from 1976, lumbar spondylolisthesis can be divided into the following five types: dysplastic, isthmic, degenerative, traumatic, and pathological (1). Among these, the most clinically common types are isthmic spondylolisthesis (IS), characterized by pars interarticularis defects (2), and degenerative spondylolisthesis (DS), which stems from disc and facet joint degeneration (3). Despite shared vertebral displacement, they exhibit significant differences in pathophysiology, clinical presentations, and radiographic features. Recently, the sagittal alignment characteristics and vertebral morphological changes of both spondylolisthesis types have been thoroughly studied (4, 5); however, the differences in coronal parameters have not received sufficient attention from surgeons.

Although often overshadowed by more prominent sagittal plane issues, coronal balance of the spine is closely related to a patient's quality of life, pain severity, and functional disability. Previous studies have indicated that patients with coronal imbalance tend to experience more severe preoperative symptoms (6). This finding has profound implications for surgical decision-making and treatment planning. Utilizing coronal parameter analysis as a valuable tool, preoperative coronal alignment assessment should be considered an important component of management for lumbar spondylolisthesis patients. Additionally, clinical observations have found that segmental scoliosis or lateral olisthesis of the lumbar spine affects the occurrence and distribution of lumbar and lower limb pain. However, existing studies mainly investigate the correlation between the Meyerding grading of spondylolisthesis and the intensity of pain (7, 8), neglecting the impact of the spinal coronal alignment on the severity and distribution of pain.

To our knowledge, no studies have yet explored the difference in spinal coronal balance between degenerative and isthmic spondylolisthesis and its correlation with clinical characteristics. Therefore, the objectives of this study are: (1) to measure the coronal radiographic parameters of DS and IS patients using the whole-spine anteroposterior radiographs; (2) to evaluate the coronal balance of the DS group and IS group, and to clarify the key distinguishing factors between the two groups; (3) to explore the correlation between coronal parameters and clinical characteristics, and to predict the effect of the segmental wedge angle (including both magnitude and direction) on the distribution of lower limb pain in the DS group by decision tree analysis.

## 2 Materials and methods

### 2.1 Study population

This retrospective cross-sectional study was performed using consecutively recruited patients from the Department of Spine Surgery, Shandong Provincial Hospital from March 2022 to December 2024. All enrolled patients had single-segment lumbar spondylolisthesis and presented with clinical symptoms such as lower back pain or lower limb pain with numbness, following a three-month period of ineffective conservative management. All participants were subjected to physical examination and digital x-ray photography of the whole spine. The inclusion criteria were as follows: (1) A definitive diagnosis of monosegmental degenerative or isthmic lumbar spondylolisthesis confirmed by two senior spine surgeons; (2) Complete radiographic datasets permitting precise parameter quantification; (3) Age ≥18 years with treatment-naïve status during observation window; (4) Clinically significant symptoms meeting the modified Meyerding classification criteria. The exclusion criteria included: (1) Multilevel lumbar spondylolisthesis; (2) Dysplastic spondylolisthesis or idiopathic spinal deformities; (3) Acute lumbar disc herniation requiring emergent intervention; (4) Previous spinal instrumentation; (5) Systemic bone diseases (such as neoplasms, spondyloarthropathies, infectious spondylitis); (6) Radiographic evidence of vertebral compression fractures, significant vertebral body wedging, or intrinsic vertebral height inequality unrelated to spondylolisthesis (to minimize the confounding effect of osteoporotic morphological changes on coronal wedge angle measurements); (7) Non-diagnostic imaging due to non-standardized radiographic positioning or motion artifacts. Based on the inclusion and exclusion criteria, a total of 150 patients met the eligibility criteria and were included in the study, of which 99 were diagnosed with DS and 51 with IS.

### 2.2 Data collection

This study collected basic clinical information, including patient name, height, weight, body mass index (BMI), age, and the degree of spondylolisthesis. Pain and functional scores were documented, including the Visual Analog Scale (VAS) for back and leg pain, as well as the Oswestry Disability Index (ODI), which ranges from 0 to 100, with higher scores indicating greater disability severity. VAS-back, VAS-leg, and ODI were collected at hospital admission as part of the routine preoperative assessment. All patient-reported outcomes were recorded before surgery and before final surgical planning. To assess the relationship between coronal imbalance and clinical symptoms, the side experiencing greater pain was recorded for each patient to facilitate analysis of the relationship between the concave or convex side of the coronal plane alignment and the area of pain distribution.

### 2.3 Imaging data

Coronal parameters were measured on standing whole-spine anteroposterior radiographs. These included the clavicle angle, pelvic coronal inclination, T1 tilt, segmental wedge angle, lumbar Cobb angle, slipped vertebral tilt angle, and lateral vertebral translation. The distance between the C7 plumb line and the central sacral vertical line (C7PL-CSVL) and lumbar coronal balance were also measured (Figure 1). All measurements were performed by one spine surgeon and one radiologist who had received the same standardized training. Both raters were blinded to the patients' diagnostic classification during measurement. When discrepancies or disagreements arose, a senior physician performed a third assessment, and the measurements were reviewed accordingly. This procedure aimed to minimize measurement bias and improve the consistency and reliability of radiographic measurements.

**Figure 1:**
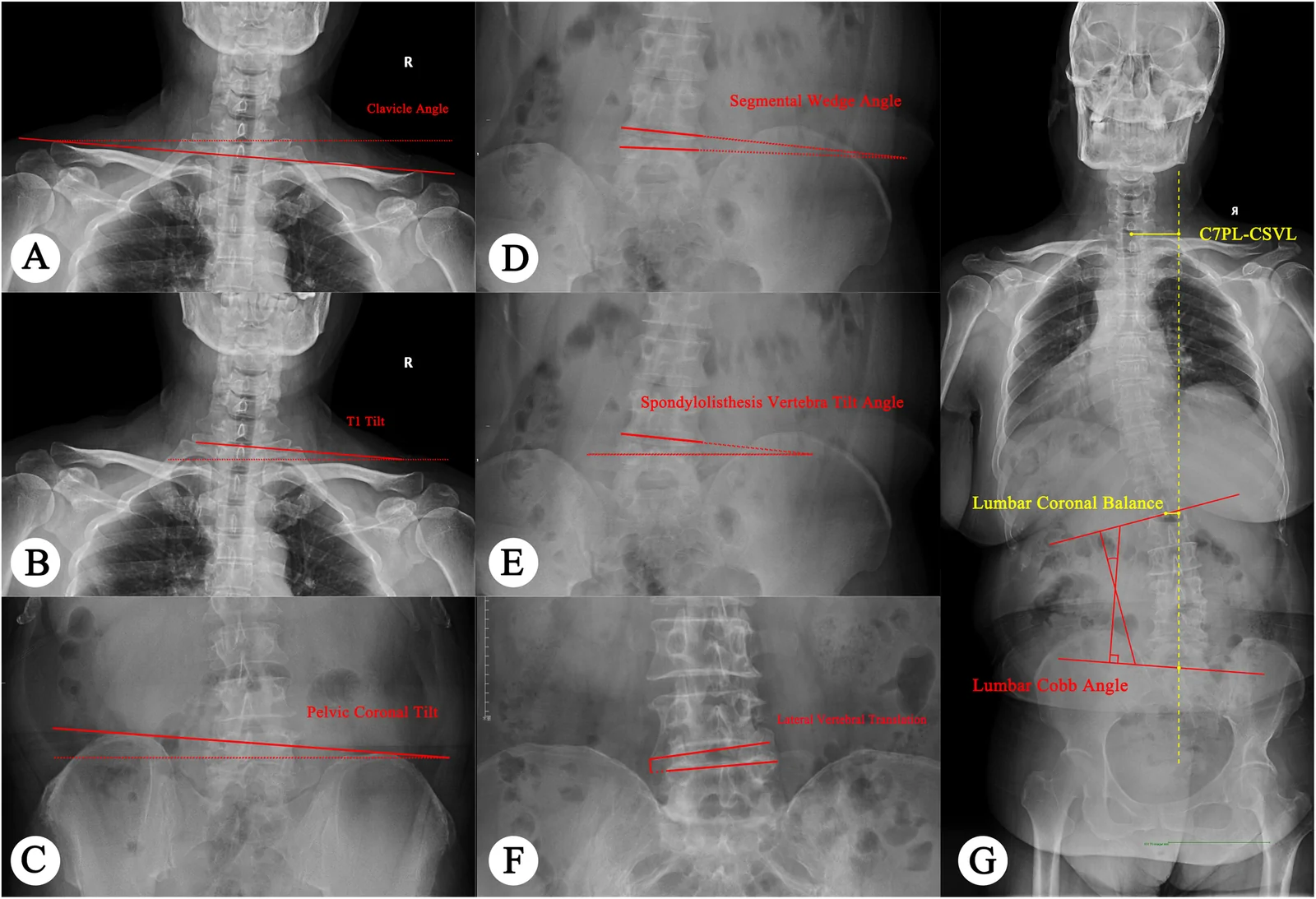
Schematic diagram of coronal spinal parameters measurement in patients with lumbar spondylolisthesis. **(A)** Clavicle angle: The angle between the line connecting the highest points of both clavicles and the horizontal reference line. **(B)** T1 tilt: The angle between the upper endplate line of the T1 vertebra and the horizontal reference line. **(C)** Pelvic coronal tilt: The angle between the line connecting the bilateral iliac crests and the horizontal reference line. **(D)** Segmental wedge angle: The angle formed between the inferior endplate of the displaced vertebra and the superior endplate of the adjacent inferior vertebra. **(E)** Spondylolisthesis vertebra tilt angle: The angle between the lower endplate of the slipped vertebra and the horizontal reference line. **(F)** Lateral vertebral translation: The horizontal distance between the intersection of a vertical line drawn downward from the left edge of the lower endplate of the slipped vertebra to the upper endplate of the subjacent vertebra, and the left edge of the upper endplate of the subjacent vertebra. **(G)** Lumbar Cobb angle: The angle between the upper endplate of L1 and the upper endplate of S1; Lumbar coronal balance: The horizontal distance between the vertical line from the midpoint of the L1 upper endplate and the central sacral vertical line (CSVL); C7PL–CSVL: The horizontal distance between the C7 plumb line and the central sacral vertical line.

Inter-observer reliability was assessed using intraclass correlation coefficients (ICCs), and the ICCs for all coronal radiographic parameters ranged from 0.79 to 0.97, indicating good to excellent measurement reliability (Supplementary Table 1).

All patients also underwent preoperative CT and MRI examinations as part of the routine diagnostic assessment. Standing radiographs were used to measure the segmental wedge angle and to classify patients into different angular groups. After this grouping, CT and MRI findings were reviewed within each group to assess neural compression-related factors, including disc herniation, lateral recess stenosis, foraminal stenosis, facet hypertrophy, ligamentum flavum hypertrophy, and the side of nerve root compression.

### 2.4 Statistical analysis

The Shapiro–Wilk test was used to assess the normality of the data distribution. Categorical data were analyzed using Pearson's chi-square test. The comparison of continuous variables was performed by independent samples t-test. All statistical analyses were performed using SPSS software (version 27; IBM Corp., Armonk, NY, USA) and R Studio version 4.4.1. A *P* value < 0.05 was considered statistically significant.

## 3 Results

### 3.1 Patient population

The baseline characteristics of the two groups are summarized in Table 1. The mean age of patients with DS was 61 ± 7 years, which was significantly higher than that of those with IS, who had a mean age of 54 ± 7 years (*P* < 0.001). Gender distribution differed significantly between the two groups (*χ*^2^ = 22.59, *P* < 0.001), with women comprising 79.8% of the DS group vs. 41.2% of the IS group. Furthermore, patients in the DS group had significantly lower mean weight (68 ± 11 kg) and height (1.6 ± 0.1 m) compared to those in the IS group (73 ± 9 kg and 1.7 ± 0.1 m, respectively; *P* < 0.01). No statistically significant difference in BMI was observed between the two groups (25 ± 3.5 vs. 26 ± 3.1; *P* = 0.137).

**Table 1:** Baseline demographic and clinical characteristics of patients with degenerative spondylolisthesis and isthmic spondylolisthesis.

Variable	Disease classification		*P value*
	Degenerative, *N* = 99	Isthmic, *N* = 51	
Age (years)	61 ± 7	54 ± 7	<0.001
Gender			<0.001
Man	20 (20.2%)	30 (58.8%)	
Woman	79 (79.8%)	21 (41.2%)	
Weight (kg)	68 ± 11	73 ± 9	0.003
Height (m)	1.6 ± 0.1	1.7 ± 0.1	0.009
BMI	25 ± 3.5	26 ± 3.1	0.137
Slipped level			
L3/4	7 (7.1%)	1 (2.0%)	
L4/5	71 (71.7%)	15 (29.4%)	
L5/S1	21 (21.2%)	35 (68.6%)	

Mean ± SD; n (%).

*P* values were calculated using Welch's two-sample t-test for continuous variables and Pearson's chi-square test for gender.

### 3.2 Spinal coronal plane parameters

No statistically significant difference was found between the DS and IS groups in terms of overall coronal spinal alignment (Clavicle angle: 2.5 ± 1.8 vs. 2.1 ± 1.7, *P* = 0.189; Pelvic coronal tilt: 2.0 ± 1.4 vs. 1.7 ± 1.4, *P* = 0.140; T1 tilt: 3.4 ± 3.0 vs. 3.2 ± 2.1, *P* = 0.768; C7PL-CSVL: 14 ± 13 vs. 11 ± 6, *P* = 0.088) (Table 2). Patients with DS demonstrated significant differences in several coronal parameters in the lumbar region compared to those with IS. Compared with IS, DS was associated with significantly greater segmental wedge angle (2.7 ± 1.9 vs. 1.8 ± 1.7; *P* = 0.005), spondylolisthesis vertebra tilt angle (4.7 ± 3.6 vs. 3.5 ± 2.4; *P* = 0.016), and worse lumbar coronal balance (10 ± 7 vs. 5 ± 3; *P* < 0.001). The intergroup differences in lumbar Cobb angle (5.6 ± 4.4 vs. 4.3 ± 4.5; *P* = 0.087) and lateral vertebral translation (3.7 ± 3.8 vs. 3.2 ± 2.6; *P* = 0.352) did not reach statistical significance.

**Table 2:** Comparative analysis of coronal plane parameters in patients with degenerative spondylolisthesis and isthmic spondylolisthesis.

Spinal Coronal Plane Parameters	Disease classification		*P value*
	Degenerative, *N* = 99	Isthmic, *N* = 51	
Clavicle angle	2.5 ± 1.8	2.1 ± 1.7	0.189
Pelvic coronal tilt	2.0 ± 1.4	1.7 ± 1.4	0.140
T1 tilt	3.4 ± 3.0	3.2 ± 2.1	0.768
Segmental wedge angle	2.7 ± 1.9	1.8 ± 1.7	0.005
Lumbar Cobb angle	5.6 ± 4.4	4.3 ± 4.5	0.087
Spondylolisthesis vertebra tilt angle	4.7 ± 3.6	3.5 ± 2.4	0.016
Lateral vertebral translation	3.7 ± 3.8	3.2 ± 2.6	0.352
C7PL-CSVL	14 ± 13	11 ± 6	0.088
Lumbar coronal balance	10 ± 7	5 ± 3	<0.001

Mean ± SD.

Welch Two Sample t-test.

To reduce the potential influence of demographic differences between the DS and IS groups, additional multivariable linear regression analyses were performed with adjustment for age, sex, height, and weight. After adjustment, segmental wedge angle remained significantly greater in the DS group than in the IS group (adjusted *β* = 0.774, 95% CI: 0.089–1.459, *P* = 0.027), and lumbar coronal balance also remained significantly different between the two groups (adjusted *β* = 4.802, 95% CI: 2.385–7.218, *P* < 0.001). Lumbar Cobb angle also showed a significant adjusted association with DS (adjusted *β* = 1.829, 95% CI: 0.161–3.498, *P* = 0.032) (Supplementary Table 2).

### 3.3 Logistic regression analysis of predictive factors for DS and IS

Multivariate logistic regression analysis determined that segmental wedge angle (OR=1.42, 95% CI: 1.07–1.88, *P* = 0.015) and lumbar coronal balance (OR=1.18, 95% CI: 1.08–1.30, *P* < 0.001) are statistically significant factors in predicting the type of spondylolisthesis (Table 3). These two factors may be important radiographic features in coronal plane that differentiate between DS and IS. In contrast, other factors including clavicle angle, pelvic coronal tilt, lumbar Cobb angle, spondylolisthesis vertebra tilt angle, and C7PL-CSVL distance, showed no significant impact (Clavicle angle: OR=1.07, 95% CI: 0.85–1.34, *P* = 0.588; Pelvic coronal tilt: OR=1.17, 95% CI: 0.86–1.58, *P* = 0.317; Lumbar Cobb angle: OR=1.07, 95% CI: 0.97–1.19, *P* = 0.178; Spondylolisthesis vertebra tilt angle: OR=0.98, 95% CI: 0.82–1.18, *P* = 0.851; C7PL-CSVL: OR=1.00, 95% CI: 0.96–1.04, *P* = 0.878). The goodness-of-fit indicators of the model, including AIC (174) and BIC (198), suggested that the model had good fit.

**Table 3:** Multivariate logistic regression was used to analyze the influencing factors of DS and IS.

Spinal Coronal Plane Parameters	N	Event N	OR	95% CI	*P* value
Clavicle angle	150	99	1.07	0.85, 1.34	0.588
Pelvic coronal tilt	150	99	1.17	0.86, 1.58	0.317
Segmental wedge angle	150	99	1.42	1.07, 1.88	0.015
Lumbar Cobb angle	150	99	1.07	0.97, 1.19	0.178
Spondylolisthesis vertebra tilt angle	150	99	0.98	0.82, 1.18	0.851
C7PL-CSVL	150	99	1.00	0.96, 1.04	0.878
Lumbar coronal balance	150	99	1.18	1.08, 1.30	<0.001

OR, Odds Ratio, CI, Confidence Interval.

Null deviance = 192; Null df = 149; Log-likelihood = −77.9; AIC = 174 BIC = 198 Deviance = 158; Residual df = 142; No. Obs. = 150.

A *post-hoc* power analysis was further performed to evaluate the statistical power for detecting intergroup differences in coronal parameters. The analysis showed that the statistical power was highest for lumbar coronal balance (99%) and close to the conventional 80% threshold for segmental wedge angle (80%). In contrast, several secondary parameters, including T1 tilt, lateral vertebral translation, and C7PL-CSVL, showed relatively limited statistical power (Supplementary Table 3).

### 3.4 VAS and ODI

The mean VAS-leg score was significantly higher in the DS group compared with the IS group (6.0 ± 1.1 vs. 4.6 ± 1.6; *P* < 0.001) (Table 4). No statistically significant differences were observed between the IS and DS groups in the VAS-back scores (5.6 ± 1.3 vs. 5.2 ± 1.3; *P* = 0.119) or in the ODI scores (61 ± 9 vs. 59 ± 10; *P* = 0.308).

**Table 4:** Comparative analysis of VAS-back, VAS-leg, and ODI scores between the DS and IS groups.

	Disease classification		*P* value
	Degenerative, *N* = 99	Isthmic, *N* = 51	
VAS-back	5.6 ± 1.3	5.2 ± 1.3	0.119
VAS-leg	6.0 ± 1.1	4.6 ± 1.6	<0.001
ODI	61 ± 9	59 ± 10	0.308

Welch Two Sample t-test.

### 3.5 The relationship between segmental wedge angle and the distribution of lower limb pain in patients with DS

Considering the higher VAS-leg scores in the DS group, we constructed a decision tree model to explore the relationship between segmental wedge angle and the prediction of lower-limb pain distribution (Figure 2). The model identified two main split points at approximately 1.3° and 5.0°. Specifically, patients with a segmental wedge angle within the 1.3°−5.0° range showed a higher prediction success rate for pain-side distribution, reaching 71.9% in the present cohort. In contrast, prediction performance was less consistent when the segmental wedge angle was <1.3° or ≥5.0°. These findings suggest that the 1.3°−5.0° range may provide a useful preliminary radiographic reference for evaluating pain-side distribution in patients with DS.

**Figure 2:**
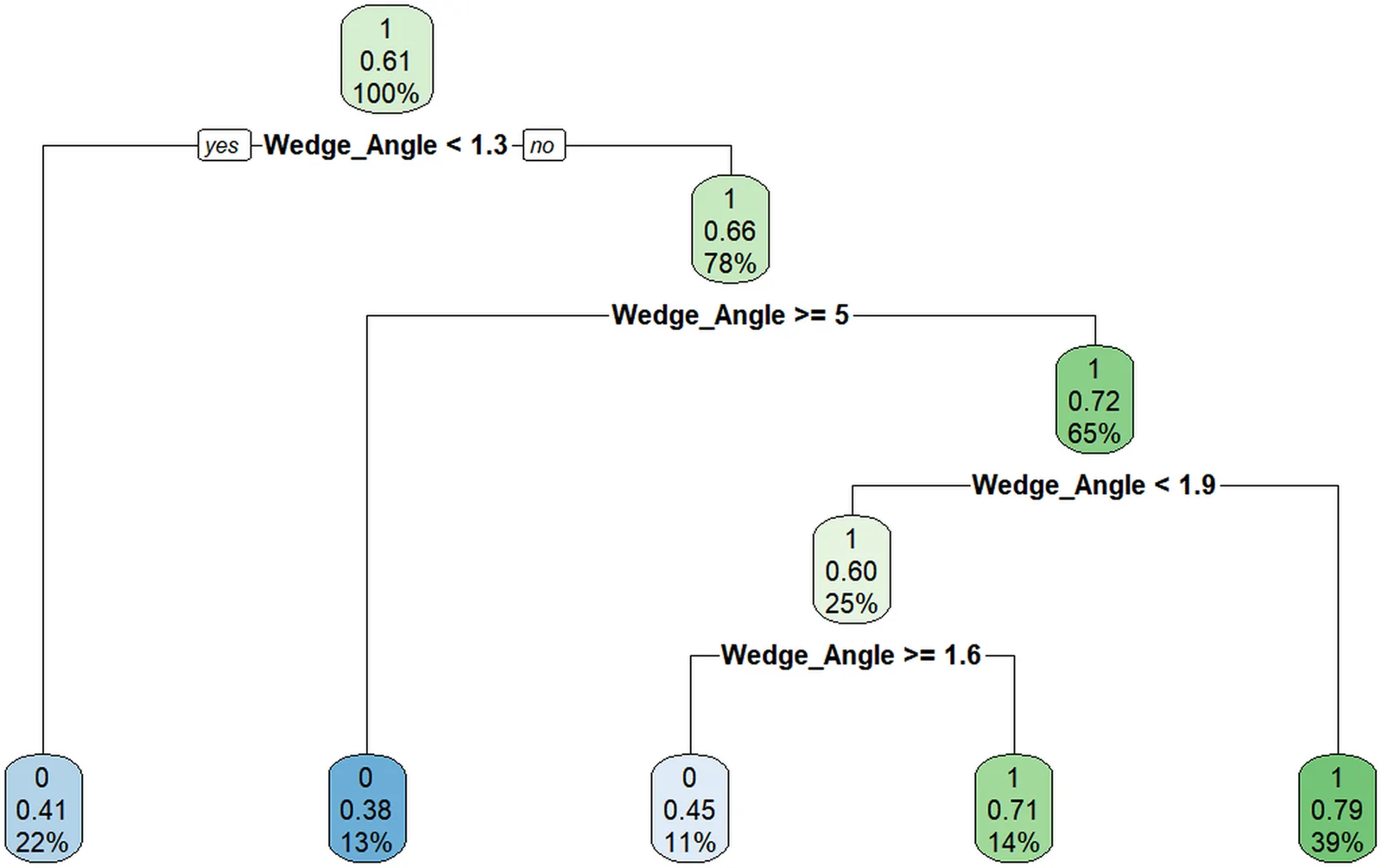
Analysis of the relationship between segmental wedge angle and pain prediction success rate based on a decision tree model built in RStudio. The model identified approximately 1.3° and 5.0° as the main split points. The 1.6° and 1.9° nodes represent secondary model-derived splits within the intermediate angular range and are not considered independent clinical thresholds. The three segmental wedge angle groups comprised 22 patients with angles <1.3°, 64 with angles ≥1.3° but <5.0°, and 13 with angles ≥5.0°. The corresponding proportions of successful pain-side predictions were 40.9% (9/22), 71.9% (46/64), and 38.5% (5/13), respectively. These results are exploratory because the split points were derived and evaluated in the same patient cohort.

To further investigate the association between segmental wedge angle and the distribution of radicular lower limb pain, a chi-square test was conducted within the range of 1.3° to 5.0° (Table 5). The test yielded a chi-square statistic of 5.3 with degrees of freedom (df = 1), corresponding to a *P* value of 0.021. These results indicate a statistically significant correlation between the segmental wedge angle (1.3°−5.0°) and the distribution of radicular pain. In other words, when the segmental wedge angle falls within the 1.3°−5.0° range, radicular pain is predominantly distributed in the lower limb corresponding to the concave side of the wedged segment (Accuracy = 0.72). When the segmental wedge angle is <1.3° or ≥5.0°, the distribution of radicular pain cannot be predicted based on the opening direction of the wedge angle.

**Table 5:** Results of chi-square test analysis.

Chi-squared	df	*P* value
5.3	1	0.021

## 4 Discussion

In this study, we confirmed the distinctions between the two types of spondylolisthesis on the coronal plane. Compared with patients with IS, those with DS exhibited larger segmental wedge angle and spondylolisthesis vertebra tilt angle, as well as more severe lumbar coronal imbalance (Figure 3). These findings highlight the distinct pathophysiological mechanisms between DS and IS. IS primarily stems from congenital defects or adolescent repetitive stress injuries (2), leading to earlier symptomatic onset and diagnosis. In contrast, DS results from age-related degenerative changes, including intervertebral disc degeneration, facet joint arthropathy, and osteoporosis (3). Individuals with greater height and weight generate larger spinal lever forces, resulting in elevated stress on the pars interarticularis and a higher risk of stress fractures. The male predominance in IS is mainly attributed to high-impact exercise and load-bearing activities, which expose the posterior column of the lumbar spine to greater biomechanical stress (6, 9).

**Figure 3:**
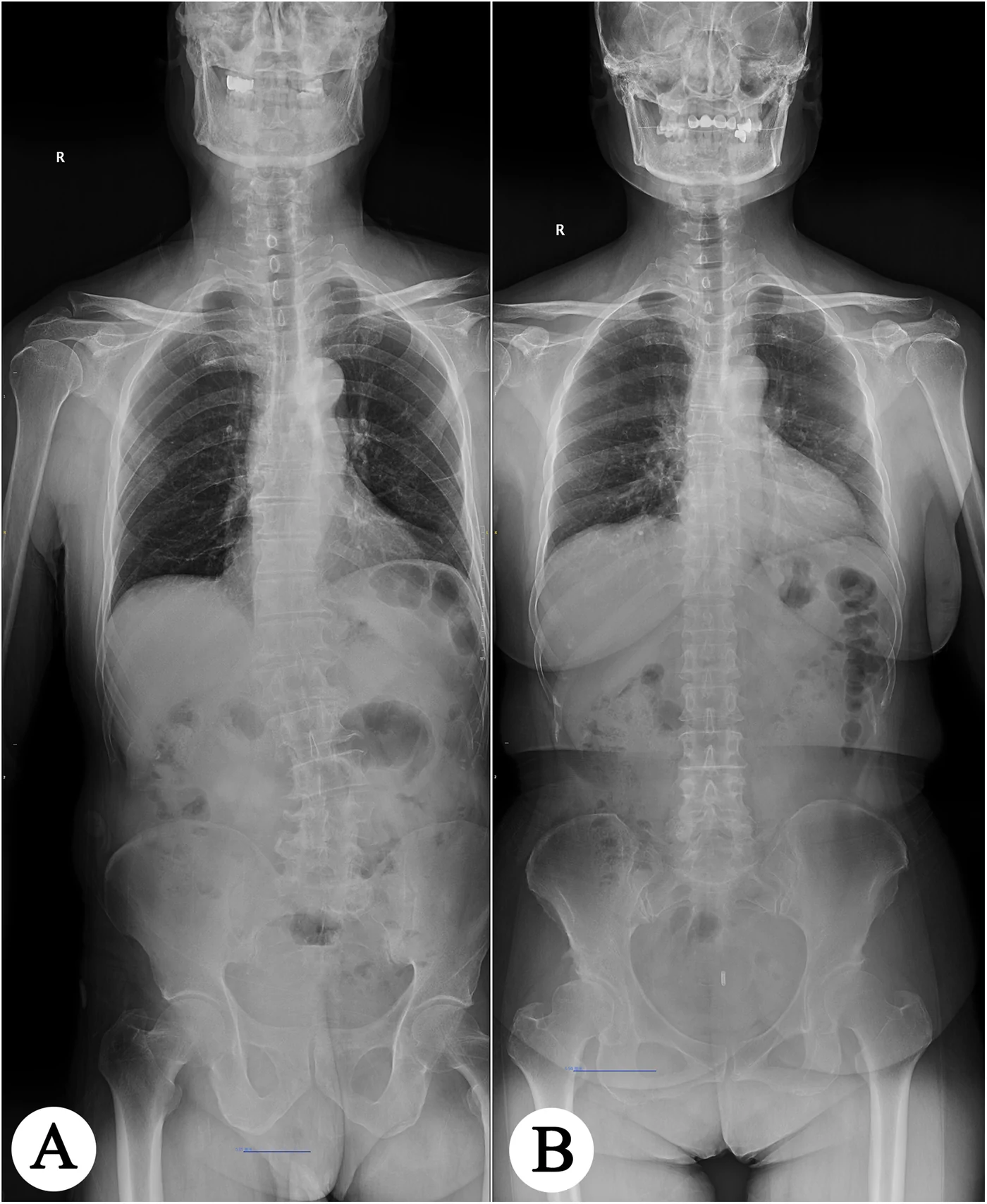
Representative anteroposterior x-ray manifestations of degenerative lumbar spondylolisthesis and isthmic spondylolisthesis of the whole spine. **(A)** Degenerative spondylolisthesis: Degenerative spondylolisthesis is characterized by lumbar scoliosis and evident coronal imbalance. Asymmetric degeneration of the intervertebral discs and facet joints leads to structural asymmetry of the lumbar spine, often resulting in coronal malalignment. **(B)** Isthmic spondylolisthesis: Isthmic spondylolisthesis is characterized by preserved vertebral alignment and largely maintained coronal balance. Because this type results from a pars defect, the slippage occurs mainly in the sagittal plane, producing only minimal coronal changes.

Previous studies of lumbar spondylolisthesis have primarily focused on sagittal alignment, and treatment strategies have evolved from simple nerve decompression to comprehensive spinopelvic sagittal correction (10, 11). In recent years, with the in-depth understanding of the causes of lumbar spondylolisthesis, the clinical relevance of coronal parameters in diagnosis and treatment has gained increasing attention. Wei et al. reported that patients with coronal imbalance exhibited reduced postoperative restoration of lumbar lordosis in the sagittal plane, supporting the concept that the coronal and sagittal planes function as an integrated biomechanical system (12). However, the differences in coronal parameters among different types of spondylolisthesis still lack sufficient research. Liu et al. compared sagittal alignment, paraspinal muscles, and other anatomical characteristics between DS and IS (13). In DS, progressive degeneration of the intervertebral discs and facet joints leads to decreased stability between adjacent vertebral bodies. This degenerative process is often asymmetric, with more pronounced deterioration on one side. When one side of the support structure collapses more than the other side, the vertebra not only slips to the ventral side, but also translates or tilts toward the more degraded side. This state results in an increased segmental wedge angle and greater lumbar coronal imbalance. In contrast, the pathogenesis of IS permits anterior translation of the vertebral body without generating lateral forces that cause coronal imbalance. The finding also indicates that the adjacent segments of DS exhibit greater stiffness and worse compensatory ability to maintain spinal balance than IS. It may be related to paravertebral muscle atrophy and facet joint degenerative hypertrophy in DS patients. On the contrary, the non-affected spinal segments of IS patients usually provide stronger compensatory potential to maintain the overall good alignment of the spine.

No significant differences were observed between DS and IS groups in parameters reflecting the overall coronal alignment of the spine, including the clavicle angle, T1 tilt, pelvic coronal tilt, and the distance between C7PL-CSVL. According to the study of Haddas et al., patients with degenerative spinal disease demonstrated nearly 2 times of compensatory efforts and balance attempts compared with healthy controls (14). Since the lesion is limited to the lower lumbar spine, compensatory sequence adjustments in the thoracic and pelvic regions may be sufficient to maintain the stability of the global coronal balance. In addition, the slow progress of DS makes the compensatory mechanism play an exact role in maintaining the overall spinal alignment (15). There was no significant difference in the lateral translation distance of the spondylolisthesis vertebra between the DS and IS groups, suggesting that the lateral olisthesis of the DS segment was limited by the coronal facet orientation.

Coronal radiographic parameters are not only indicators of spinal balance, but also significantly correlated with the clinical characteristics of patients (16, 17). In this study, the separate VAS scores of low back pain and leg pain revealed that DS patients primarily presented with low back pain and radicular lower limb pain, whereas IS patients mainly experienced localized low back pain. DS is often accompanied by ligamentum flavum hypertrophy, facet joint hyperosteogeny and spinal canal stenosis, which in turn causes mechanical compression of the nerve roots. The segmental instability of IS triggers sustained compensatory contraction of the paraspinal muscles and related ligaments, which eventually leads to chronic low back pain (18–20). Compared with DS, IS is usually associated with milder disc degeneration and wider intervertebral foramina, thereby reducing the likelihood of nerve root compression (21).

To further investigate the potential causes of severe lower limb pain in the DS group, we performed a decision tree analysis to predict the effect of the wedge angle of the spondylolisthesis segment, including magnitude and direction, on the distribution of lower limb pain. Lateral vertebral translation was not included as the primary variable in the initial decision tree model because the model was primarily designed to explore the association between segmental wedge angle and pain-side distribution. The results showed that when the wedge angle was in the range of 1.3° to 5.0°, the opening direction of segmental scoliosis had a strong correlation with the distribution of lower limb pain. When the segmental wedge angle was less than 1.3°, the local coronal alignment remained essentially normal, and there was little difference in traction or asymmetric mechanical compression between the bilateral nerve roots. In this state, the correlation between the area of lower limb pain and the direction of the segmental wedge angle was weak, with symptoms more likely attributable to pre-existing compression factors, such as disc herniation or lateral recess stenosis. As the segmental wedge angle increases (1.3°–5.0°), the biomechanical alterations lead to greater impingement between the inferior and superior articular processes on the concave side, resulting in osteophyte formation and ligamentum flavum hypertrophy. In severe angular deformities, the concave inferior articular process may even subluxate anteriorly and strike the traversing nerve root, thereby exacerbating radicular pain. In this state, radicular pain mainly distributed in the lower limb on the concave side of the wedged segment. We found that the side of leg pain corresponded to the side of nerve root compression shown on CT/MRI. This correspondence may be related to asymmetric neural compression associated with segmental coronal angulation and accompanying facet joint hypertrophy or subluxation. When the segmental wedge angle exceeded 5.0°, traction on the nerve root on the convex side was observed to surpass the physiological threshold, which may result in nerve root injury (22). Meanwhile, the widening of the facet joint space and dorsal tilting of the inferior articular process may stimulate osteophyte formation at the convex facet joint, leading to the lateral recess stenosis on the convex side (23, 24). Therefore, within this angular range, the correlation between the direction of the wedge angle and the distribution of radicular pain appeared to lack specificity (Figure 4). The range of 1.3° −5.0° has an average prediction accuracy of 72%. In the failure prediction set, more than half of the patients showed a lateral vertebral translation of more than 3 mm (Figure 5), which may have further increased traction on the convex-side nerve root and reduced the prediction accuracy of the segmental wedge angle. These findings indicate that the relationship between segmental wedge angle and pain-side distribution may vary across different angular ranges, with the 1.3°−5.0° range showing relatively better predictive consistency in the present cohort.

**Figure 4:**
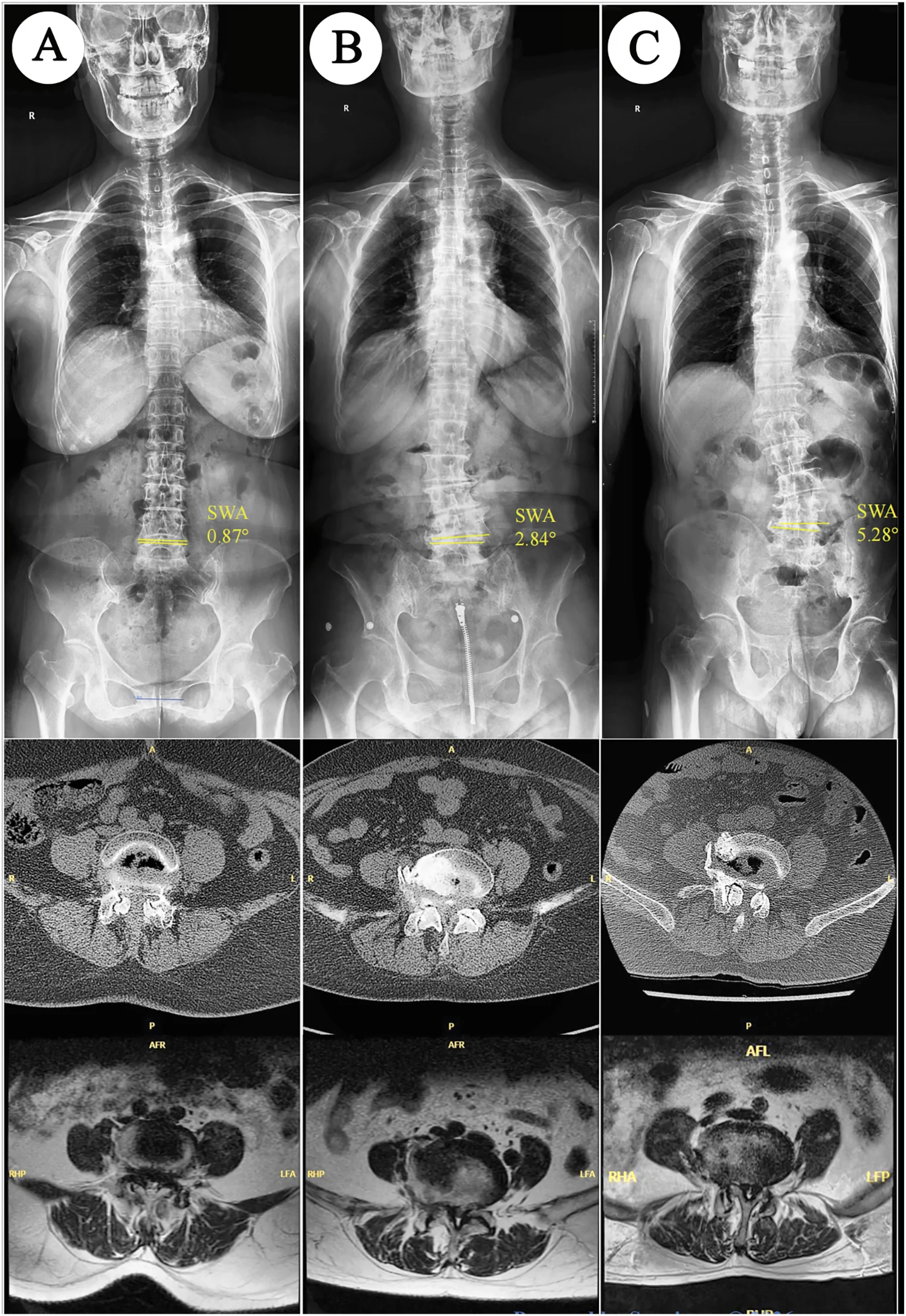
Representative images showing the compression status of nerve roots on the concave and convex sides with varying magnitudes of segmental wedge angles. **(A)** corresponds to a segmental wedge angle < 1.3°, Fig B to 1.3°-5.0°, and Fig C to > 5.0°.

**Figure 5:**
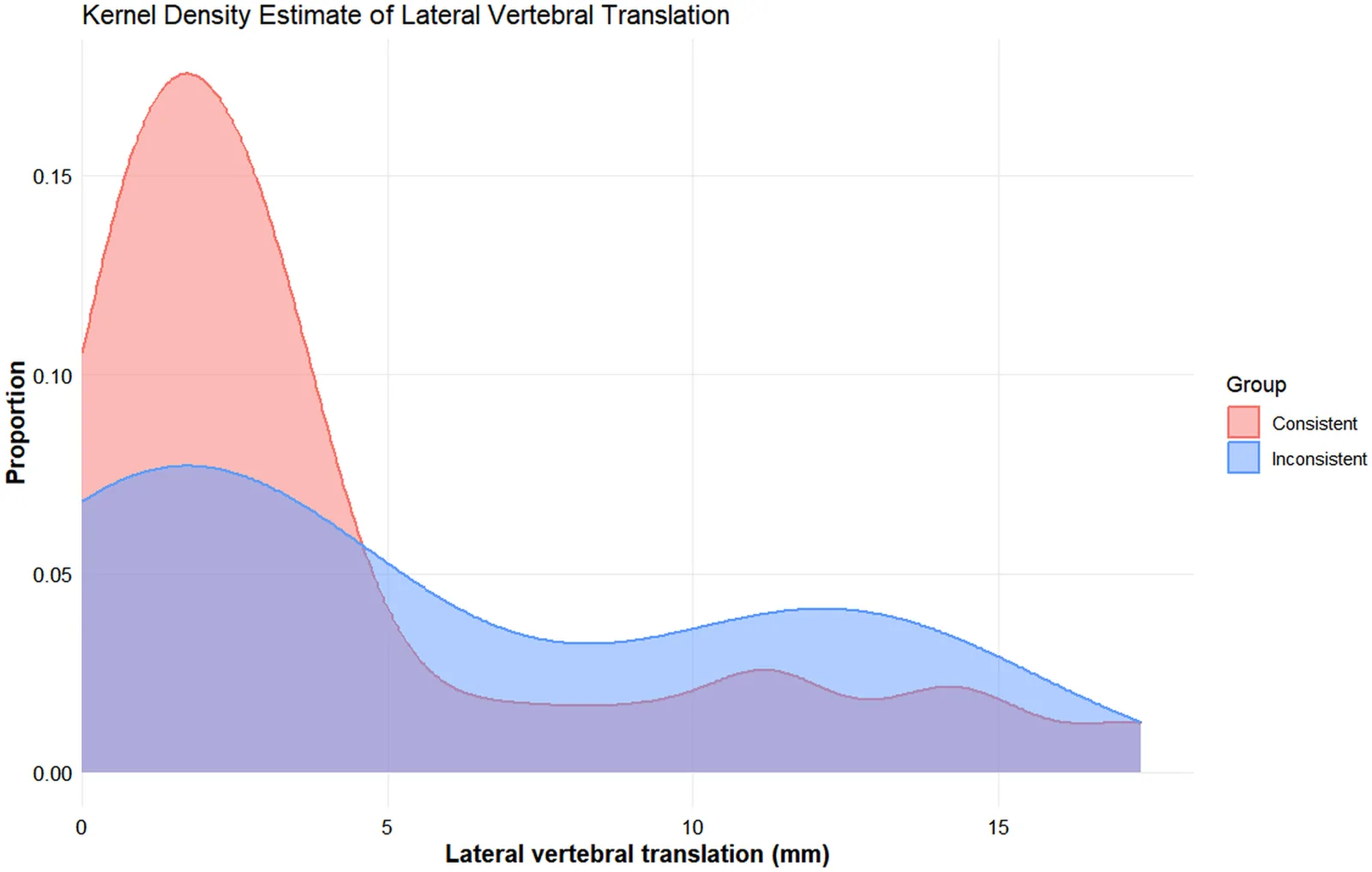
Effect of lateral vertebral translation on prediction when the segmental wedge angle is between 1.3° and 5.0°.

Historically, similar surgical strategies have been applied to the management of DS and IS (25–27). The present findings provide additional insight into the relationship between coronal segmental morphology and radicular lower-limb pain distribution in patients with DS. From an exploratory clinical perspective, the segmental wedge angle may serve as a preliminary radiographic reference during preoperative evaluation and surgical planning. When the wedge angle of the spondylolisthetic segment is within the range of 1.3°−5.0°, surgeons may pay closer attention to the possible relationship between coronal segmental morphology and concave-side neural compression. When the wedge angle exceeds 5.0°, neural compression on both the concave and convex sides should be carefully evaluated. However, these observations should be interpreted together with CT, MRI, clinical symptoms, and physical examination findings, rather than being used as independent criteria for surgical decompression. Further prospective studies are needed to validate the clinical applicability of these findings.

Our study also has several limitations. First, this was a retrospective single-center study with a relatively limited sample size, particularly in the IS group. *Post-hoc* power was low for several secondary coronal parameters, so small between-group differences may have been missed. The DS and IS groups were also unevenly represented within each slip level, with only one IS patient at L3/4. These small, imbalanced subgroups limited the precision of level-adjusted estimates and within-level comparisons. Second, although demographic differences existed between the DS and IS groups, additional multivariable analyses were performed to adjust for age, sex, height, and weight. The main coronal differences, particularly segmental wedge angle and lumbar coronal balance, remained significant after adjustment. Nevertheless, residual confounding may still exist because of the retrospective design and the inherent epidemiological differences between DS and IS. Finally, the present study primarily focused on coronal radiographic morphology. Sagittal parameters, such as pelvic incidence, lumbar lordosis, sagittal vertical axis, and slip severity, may interact with coronal compensation, but were not systematically incorporated into the current analysis. Future multicenter prospective studies with larger and more balanced cohorts should further validate these findings and clarify the three-dimensional compensatory mechanisms in lumbar spondylolisthesis.

## 5 Conclusions

Coronal balance is an important parameter that should be carefully considered in the evaluation and management of patients with lumbar spondylolisthesis. DS is typically characterized by more severe lumbar coronal imbalance and larger segmental wedge angle. The coronal wedge angle of the spondylolisthesis segment serves as a key parameter to distinguish DS from IS. When it is within the range of 1.3° to 5.0°, its opening direction is found to be closely related to the distribution of lower limb pain in DS patients, indicating that the concave and convex sides of the segmental angle have an impact on clinical characteristics.

## Data Availability

The original contributions presented in the study are included in the article/Supplementary Material, further inquiries can be directed to the corresponding authors.
