# Supplementary material for: Comparative analysis of coronal balance with clinical correlation between degenerative and isthmic lumbar spondylolisthesis: a retrospective study
Source: Front Med (Lausanne). 2026 Oct 23;13:1902310. doi: 10.3389/fmed.2026.1902310 (PMC13739589; doi:10.3389/fmed.2026.1902310)
Supplement: Supplementary file 1 [file Table1.docx]

| **Parameters** | **Inter-observer ICC** |
| --- | --- |
|  |  |
| Clavicle angle | 0.89 |
| Pelvic coronal tilt | 0.90 |
| T1 tilt | 0.88 |
| Segmental wedge angle | 0.93 |
| Lumbar Cobb angle | 0.97 |
| Spondylolisthesis vertebra tilt angle | 0.94 |
| Lateral vertebral translation | 0.87 |
| C7PL-CSVL | 0.79 |
| Lumbar coronal balance | 0.85 |

Supplementary Table 1：Inter-observer reliability of coronal radiographic measurements
