# Supplementary material for: Comparative analysis of coronal balance with clinical correlation between degenerative and isthmic lumbar spondylolisthesis: a retrospective study
Source: Front Med (Lausanne). 2026 Oct 23;13:1902310. doi: 10.3389/fmed.2026.1902310 (PMC13739589; doi:10.3389/fmed.2026.1902310)
Supplement: Supplementary file 2 [file Table2.docx]

| Parameter | Degenerative | Isthmic | Unadjusted P value | Adjusted β for DS vs IS | 95% CI | Adjusted P value | N |
| --- | --- | --- | --- | --- | --- | --- | --- |
| Clavicle angle | 2.51 ± 1.84 | 2.11 ± 1.73 | 0.189 | 0.164 | -0.518 to 0.847 | 0.635 | 150 |
| Pelvic coronal tilt | 2.04 ± 1.44 | 1.68 ± 1.44 | 0.140 | 0.371 | -0.180 to 0.921 | 0.185 | 150 |
| T1 tilt | 3.35 ± 2.95 | 3.23 ± 2.12 | 0.768 | 0.605 | -0.405 to 1.614 | 0.238 | 150 |
| Segmental wedge angle | 2.65 ± 1.86 | 1.78 ± 1.67 | 0.005 | 0.774 | 0.089 to 1.459 | 0.027 | 150 |
| Lumbar Cobb angle | 5.62 ± 4.35 | 4.30 ± 4.48 | 0.087 | 1.829 | 0.161 to 3.498 | 0.032 | 150 |
| Spondylolisthesis vertebra tilt angle | 4.69 ± 3.63 | 3.48 ± 2.39 | 0.016 | 1.201 | -0.051 to 2.454 | 0.060 | 150 |
| Lateral vertebral translation | 3.67 ± 3.76 | 3.18 ± 2.57 | 0.352 | 0.775 | -0.519 to 2.069 | 0.239 | 150 |
| C7PL-CSVL | 13.74 ± 12.85 | 11.07 ± 6.21 | 0.088 | 4.147 | -0.109 to 8.403 | 0.056 | 150 |
| Lumbar coronal balance | 10.07 ± 7.38 | 5.25 ± 3.38 | <0.001 | 4.802 | 2.385 to 7.218 | <0.001 | 150 |

Supplementary Table 2. Multivariable-adjusted comparison of coronal radiographic parameters between the DS and IS groups
