# Supplementary material for: Comparative analysis of coronal balance with clinical correlation between degenerative and isthmic lumbar spondylolisthesis: a retrospective study
Source: Front Med (Lausanne). 2026 Oct 23;13:1902310. doi: 10.3389/fmed.2026.1902310 (PMC13739589; doi:10.3389/fmed.2026.1902310)
Supplement: Supplementary file 3 [file Table3.docx]

| **Parameter** | **Degenerative** | **Isthmic** | **Cohen d** | **Post-hoc power (%)** |
| --- | --- | --- | --- | --- |
| Clavicle angle | 2.51 ± 1.84 | 2.11 ± 1.73 | 0.223 | 25 |
| Pelvic coronal tilt | 2.04 ± 1.44 | 1.68 ± 1.44 | 0.256 | 32 |
| T1 tilt | 3.35 ± 2.95 | 3.23 ± 2.12 | 0.046 | 6 |
| Segmental wedge angle | 2.65 ± 1.86 | 1.78 ± 1.67 | 0.48 | 80 |
| Lumbar Cobb angle | 5.62 ± 4.35 | 4.30 ± 4.48 | 0.3 | 41 |
| Spondylolisthesis vertebra tilt angle | 4.69 ± 3.63 | 3.48 ± 2.39 | 0.371 | 58 |
| Lateral vertebral translation | 3.67 ± 3.76 | 3.18 ± 2.57 | 0.143 | 13 |
| C7PL-CSVL | 13.74 ± 12.85 | 11.07 ± 6.21 | 0.242 | 29 |
| Lumbar coronal balance | 10.07 ± 7.38 | 5.25 ± 3.38 | 0.762 | 99 |

Supplementary Table 3. Post-hoc power analysis for intergroup comparisons of coronal radiographic parameters
